# Association between retinal vein occlusion, axial length and vitreous chamber depth measured by optical low coherence reflectometry

**DOI:** 10.1186/s12886-015-0031-1

**Published:** 2015-04-30

**Authors:** Andrea Szigeti, Miklós Schneider, Mónika Ecsedy, Zoltán Zsolt Nagy, Zsuzsanna Récsán

**Affiliations:** Department of Ophthalmology, Semmelweis University, 39 Mária Str, 1085 Budapest, Hungary

**Keywords:** Retinal vein occlusion, Central retinal vein occlusion, Branch retinal vein occlusion, Axial length, Vitreous chamber depth, Optical low coherence reflectometry

## Abstract

**Background:**

Results of ocular biometric measurements in retinal vein occlusion (RVO) eyes are still inconclusive and controversial. The aim of this study was to evaluate the association between ocular axial length (AL), vitreous chamber depth (VCD) and both central (CRVO) and branch retinal vein occlusions (BRVO) using optical low coherence reflectometry (OLCR).

**Methods:**

Both eyes of 37 patients with unilateral CRVO (mean age: 66 ± 14 years, male:female - 21:16) and 46 patients with unilateral BRVO (mean age: 63 ± 12 years, male:female - 24:22) were enrolled in this study. The control group consisted of randomly selected single eyes of 67 age and gender matched volunteers without the presence or history of RVO (mean age: 64 ± 14 years, male:female - 34:33). Optical biometry was performed by OLCR biometer (LenStar LS 900). Average keratometry readings, central corneal thickness (CCT), anterior chamber depth (ACD), lens thickness (LT), AL and VCD of eyes with RVO were compared with those of fellow eyes using paired t-tests and with those of control eyes using independent t-tests.

**Results:**

Mean CCT, ACD and LT, average keratometry readings of affected RVO eyes, unaffected fellow eyes and control eyes was not statistically different in either groups. In eyes with CRVO mean AL and VCD of affected eyes were significantly shorter than those of control eyes (p < 0.001, p < 0.05), mean difference in AL and VCD between the affected and control eyes was 0.56 ± 0.15 mm and 0.45 ± 0.19 mm, respectively. In eyes with BRVO, mean AL of the affected eyes was significantly shorter with a mean difference of 0.57 ± 0.15 mm (p < 0.001) and the VCD was significantly shorter with a mean difference of 0.61 ± 0.15 mm (p < 0.001) comparing with the control eyes.

**Conclusion:**

Shorter AL and VCD might be a potential anatomical predisposing factor for development either of CRVO or BRVO.

## Background

Retinal vein occlusion (RVO) is the most common form of retinal vascular disease following diabetic retinopathy, and may result in permanent vision loss [1]. The Beaver Dam Eye Study [2] reported a 5-year cumulative incidence of central retinal vein occlusion (CRVO) of 0.1-0.2%. For a branch retinal vein occlusion (BRVO) this was approximately three times more at 0.6%. Many systemic and local factors can predispose to the development of RVO, including hypertension, diabetes mellitus, hyperviscosity, hyperlipidemia and primary open angle glaucoma [1,3-6].

Results of ocular biometric measurements in RVO eyes are still inconclusive and controversial [7-21]. Some authors found association between shorter axial length (AL) or hyperopia and RVO [7-10,14,15,17,18]. In previous studies, A-scan ultrasonography and partial coherence laser interferometry were performed to measure AL in eyes with RVO (Table 1) [9-21]. Since A-scan ultrasonography (US) measures AL from the anterior cornea to the internal limiting membrane, significantly shorter AL in RVO eyes compared to control eyes reported in these studies was proposed to be due to complications from RVO (such as macular edema) [20].

**Table 1:** **Summary of previous studies using AL measurements in patients with CRVO, BRVO or hemispheric retinal vein occlusion (HRVO) based on A scan ultrasonography (** ^**†**^ **) or partial coherence laser interferometry (** ^**††**^ **) measurements** [9-21]

**Studies (first author, year of publication, AL measuring method)**	**Type of RVO**	**Number of patients with RVO/control eyes**	**Difference in AL between affected RVO and unaffected fellow eyes (mm)**	**Difference in AL between affected RVO and control eyes (mm)**
Ariturk (1996) [9]^†^	BRVO	41/66	0.10	0.33*
Timmerman (1997) [10]^†^	BRVO	24/24	0.04	0.6*
Simons (1997) [11]^†^	BRVO	36/36	-	0.07
Bandello (1998) [12]^†^	BRVO	88/50	-	1.03
Kir (1998) [13]^†^	BRVO	50/45	0.02	0.03
Cekic (1999) [14]^†^	BRVO	27/17	0.13	0.37
Tsai (2003) [15]^†^	BRVO	77/67	0.19*	0.85*
Goldstein (2004) [16]^†^	BRVO	24	0.21*	-
Mehdizadeh (2005) [17]^†^	BRVO	18/18	0.25*	1.25**
Brown (1990) [18]^†^	CRVO	24/44	0.03	0.67*
Ariturk (1996) [9]^†^	CRVO	17/66	0.36*	0.97**
Kir (1998) [13]^†^	CRVO	39/45	0.07	0.10
Kir (1998) [13]^†^	HRVO	13/45	0.15	0.19
Bandello (1998) [12]^†^	CRVO	58/50	-	0.27
Cekic (1999) [14]^†^	CRVO	19/25	0.62*	0.95*
Tsai (2003) [15]^†^	CRVO	40/67	0.26	0.76*
Mehdizadeh (2005) [17]^†^	CRVO	18/18	0.52*	1.06*
Mirshahi (2005) [19]^†^	CRVO	30/29	0.02	0.23
Moghimi (2007) [20]^††^	CRVO	29	0.07	-
Gupta (2010) [21]^†^	RVO	25/25	0.83*	-

Asterisk indicates a difference that is considered statistically significant (*p < 0.05, **p < 0.001). ( − = not studied).

Optical low coherence reflectometry (OLCR) provides high-resolution non-contact measurements of ocular biometry using superluminescent diode light source that measures the ocular AL as a distance from anterior corneal surface to the retinal pigment epithelium, meaning that measurements of AL are unaffected by the presence of macular edema. In addition to AL, keratometric readings and anterior chamber depth (ACD), the OLCR biometer is capable of determining the central corneal thickness (CCT) and lens thickness (LT) as well [22]. Using these data vitreous chamber depth (VCD) can be calculated.

The purpose of this study was to evaluate the association between AL, VCD and RVO using ruling out measurement altering effects of macular edema.

## Methods

This prospective controlled study was performed at the Department of Ophthalmology, Semmelweis University, Budapest, Hungary. All participants were treated in accordance with the tenets of the Declaration of Helsinki. Institutional Review Board approval was obtained for all study protocols (Semmelweis University Regional and Institutional Committee of Sciences and Research Ethics). Written informed consent was obtained from all participants in this study.

Thirty seven patients with unilateral CRVO (mean age: 66 ± 14 years, range: 36–92 years, male:female - 21:16) and 46 patients with unilateral BRVO (mean age: 63 ± 12 years, range: 40–86 years, male:female - 24:22) were enrolled consecutively as they were referred to the outpatient clinic of the department for examination during the last 2 years.

The control group consisted of 67 eyes of 67 randomly selected age and gender matched volunteers (mean age: 64 ± 14 years, range: 38–90 years, male:female - 34:33). Right eye was randomly selected in 34 patients and left eye in 33 patients.

Mean duration of RVO symptoms was 5.6 months (range: 1–10 months) in CRVO group and 4.8 months (range: 1–8 months) in BRVO group. We defined ischemic type as area of retinal capillary non perfusion greater than 10 disc area in eyes with CRVO and 5 disc area in BRVO [23]. In the CRVO group, right eye was affected in 19 patients and left eye in 18 patients. Ten patients had ischemic and 27 had non-ischemic CRVO. In the BRVO group, right eye was affected in 20 patients and left eye in 26 patients. 31 patients had superotemporal BRVO and 15 had inferotemporal BRVO, 14 patients had ischemic and 32 patients had non-ischaemic BRVO.

Patients with a history of previous intraocular surgery, eye trauma, any other retinal or neurological disease (e.g. multiple sclerosis), intraocular inflammation or tumor, or significant ocular media opacities including dense cataract that precluded the optical AL measurements were excluded from the study. Exclusion criteria were the same for control participants with the addition of presence or history of RVO.

All patients with RVO underwent systemic examinations, including fasting blood glucose level determination, systemic blood pressure measurement and detailed cardiovascular and hematological examination.

Ophthalmic examination included best corrected visual acuity (BCVA, measured with Snellen chart adjusted at 5 m, converted to logMAR values for analysis), subjective spherical equivalent refraction (SER), slit lamp biomicroscopy, gonioscopy, intraocular pressure measurement with applanation tonometry, indirect ophthalmoscopy following pupil dilation and fundus fluorescein angiography in RVO patients.

Optical biometry of the eyes was performed by the LenStar LS 900 device (LS 900® Haag-Streit AG, Koeniz, Switzerland, software version: V1.3.0) which is based on the principles of OLCR. The instrument uses a broadband superluminescent diode light source (peak wavelength 820 nm) to provide a series of axial biometric dimensions along the visual axis [22,24,25]. The measurement wavelength and bandwidth of the instrument equate to an axial resolution of ~ 10 microns, using the formulas from Tanna et al. [26] For a single measurement, the instrument performs 16 consecutive scans with each measurement taking ~3-5 seconds [25]. A minimum of 5 measurements were obtained for every parameter in each eye for calculating mean values. All measurements were performed by the same operator who was masked to the subject’s eye condition.

The OLCR device measures ocular AL as a distance from the peak of anterior corneal surface to the central retinal pigment epithelium peak. Keratometry (flat and steep keratometry), central corneal thickness (CCT), anterior chamber depth (ACD- length from corneal epithelium to anterior lens surface), lens thickness (LT), and AL are automatically derived ocular biometric measures from the instrument. Average keratometric power (K_average_) was calculated as the mean of the flat and the steep keratometric readings. SER was defined as the spherical power plus half of the minus cylindrical power (sphere + ½ cylinder). VCD was defined as AL minus ACD (including CCT) and LT.

Statistical analyses were performed using SPSS software program (Statistical Package for Social Sciences, SPSS version 22.0; SPSS Inc., Chicago, IL, USA). P value of <0.05 was considered statistically significant. Differences between demographic data of RVO patients and control group were assessed by Chi-square test for categorical variables (gender, presence of hypertension and diabetes mellitus) and one-way univariate analysis of variance (ANOVA) for continuous variables (age). The distributions of K_average_, SER, CCT, ACD, LT, AL, VCD were confirmed as normally distributed by Kolmogorov-Smirnov tests and therefore K_average_, SER, CCT, ACD, LT, AL, VCD of the eyes with RVO (BRVO or CRVO) were compared with those of the unaffected fellow eyes using paired t-test. K_average_, SER, CCT, ACD, LT, AL, VCD of the affected and unaffected fellow eyes of patients with RVO (BRVO or CRVO) were compared with those of the control eyes using independent t-test.

## Results

The characteristics of the patients are summarized in Table 2. No significant differences were observed between the groups in terms of age, sex and disease factors, including diabetes mellitus and hypertension (p > 0.05).

**Table 2:** **Patients characteristics**

**Variables**	**CRVO**	**BRVO**	**Control**
**Number**	37	46	67
**Gender (male:female)** ^**†**^	21:16	24:22	34:33
**Age (mean ± SD, years)** ^**††**^	66 ± 14	63 ± 12	64 ± 14
**Hypertension (n, %)** ^**†**^	23 (62.16%)	29 (63.04%)	41 (61.19%)
**Diabetes mellitus (n, %)** ^**†**^	6 (16.22%)	7 (15.21%)	10 (14.93%)

Chi-squared test was used for categorical variables (^†^) and one-way ANOVA for continuous variables (^††^).

In the CRVO group, mean BCVA was +0.70 ± 0.63 logMAR in the affected eyes and +0.11 ± 0.36 logMAR in the unaffected fellow eyes. In the BRVO group, mean BCVAs of affected and fellow eyes were +0.41 ± 0.40 logMAR and +0.06 ± 0.14 logMAR, respectively. BCVA of control eyes was +0.11 ± 0.22 logMAR.

Tables 3 and 4 show the average group ocular biometry measurements. Comparisons were performed for SER, K_average_, CCT, ACD, LT, AL and VCD among control eyes and affected, unaffected fellow eyes in both RVO groups, respectively. Mean SER, K_average_, CCT, ACD and LT of affected eyes (both CRVO and BRVO groups), unaffected fellow eyes and control eyes were not statistically different between groups. Mean AL and VCD of affected eyes in the CRVO group were significantly shorter than those of the control eyes (p = 0.001, p < 0.05), mean difference was 0.56 ± 0.15 mm in AL and 0.45 ± 0.19 mm in VCD. The mean AL and VCD of affected eyes in CRVO patients were significantly shorter than those of the unaffected fellow eyes. (p < 0.001, p = 0.013)

**Table 3:** **Ocular biometric measurements (mean ± standard deviation) of the affected and unaffected fellow eyes in CRVO and control eyes**

	**Control eyes**	**CRVO**		**p value**		
		**Affected eyes**	**Fellow eyes**	**Affected vs control** ^**†**^	**Fellow vs control** ^**†**^	**Affected vs fellow** ^**††**^
**SER (D)**	0.51 ± 2.22	0.58 ± 2.12	0.53 ± 2.53	0.198	0.971	0.558
**K**_**average**_ **(D)**	43.24 ± 1.46	43.63 ± 1.43	43.65 ± 1.41	0.892	0.190	0.686
**CCT (μm**)	556.43 ± 43.20	553.76 ± 28.47	552.31 ± 30.30	0.706	0.630	0.684
**ACD (mm)**	3.11 ± 0.30	3.06 ± 0.43	3.06 ± 0.38	0.549	0.493	0.955
**LT (mm)**	4.29 ± 0.37	4.33 ± 0.39	4.46 ± 0.41	0.600	0.081	0.068
**AL (mm)**	23.45 ± 0.69	22.89 ± 0.89	23.28 ± 0.88	0.001*	0.301	0.000*
**VCD (mm)**	16.03 ± 0.65	15.59 ± 1.06	15.74 ± 0.85	0.022*	0.074	0.013*

P-values of paired (^††^) and independent (^†^) t test. Asterisk (*) indicates a difference that is considered statistically significant (p < 0.05).

**Table 4:** **Ocular biometric measurements (mean ± standard deviation) of the affected and unaffected fellow eyes in BRVO and control eyes**

	**Control eyes**	**BRVO**		**p value**		
		**Affected eyes**	**Fellow eyes**	**Affected vs control** ^**†**^	**Fellow vs control** ^**†**^	**Affected vs fellow** ^**††**^
**SER (D)**	0.51 ± 2.22	0.98 ± 1.79	1.26 ± 1.75	0.253	0.068	0.232
**K**_**average**_ **(D)**	43.24 ± 1.46	43.58 ± 1.29	43.69 ± 1.15	0.219	0.105	0.349
**CCT (μm)**	556.43 ± 43.20	551.87 ± 43.20	557.09 ± 39.94	0.565	0.936	0.121
**ACD (mm)**	3.11 ± 0.30	3.18 ± 0.44	3.12 ± 0.43	0.421	0.942	0.447
**LT (mm)**	4.29 ± 0.37	4.38 ± 0.44	4.39 ± 0.42	0.201	0.177	0.949
**AL (mm)**	23.45 ± 0.69	22.88 ± 0.93	22.91 ± 0.95	0.000*	0.001*	0.375
**VCD (mm)**	16.03 ± 0.65	15.43 ± 0.99	15.68 ± 0.89	0.000*	0.000*	0.709

P-values of paired (^††^) and independet (^†^) t test. Asterisk (*) indicates a difference that is considered statistically significant (p < 0.05).

There was no statistically significant difference between the affected and unaffected fellow BRVO eyes. Mean AL and VCD of the affected and unaffected fellow eyes in the BRVO group were significantly shorter than those of the control eyes (p < 0.001). AL of the affected eyes was shorter with a mean difference of 0.57 ± 0.15 mm and VCD was shorter with a mean difference of 0.61 ± 0.15 mm comparing with the control eyes. Mean AL of the unaffected fellow eyes was shorter than the control eyes with 0.53 ± 0.16 mm and VCD was shorter with a mean difference of 0.35 ± 0.16 mm.

## Discussion

In this study we investigated biometry of eyes with retinal vein occlusion using optical low coherence reflectometry. We found shorter axial and vitreous chamber depth in both CRVO and BRVO eyes compared to age and gender matched control eyes.

Demographic data and proportion of well-known risk factors (i.e. hypertension and diabetes mellitus) in our RVO patients were consistent with previous reports in the literature [3-6].

The role of AL in CRVO and BRVO patients is still controversial (Table 1) [9-21]. Some studies did not find differences in AL in eyes with CRVO [12,13,19,20] or BRVO [11-14] compared with unaffected fellow or control eyes. In contrast to these studies, others found significantly shorter AL in the affected eyes of patients with CRVO [9,14,15,17,18] or with BRVO [9,10,15,17] compared with control eyes, similar to the present study.

In most previous studies [9-19,21], ocular AL was measured by A-scan ultrasonography, which measures echo time to determine intraocular distances. The OLCR biometer has some advantages over conventional ultrasound. Clinical resolution of A-scan US in AL measurement was reported to be lower than OLCR. Using a typical 10-MHz transducer, it has a longitudinal resolution of 200 microns and a clinical accuracy of 100 to 120 microns compared to 12 microns for AL measurements by OLCR [22,27,28].

With OLCR biometry more accurate AL measurements are obtained compared to applanation ultrasound, which inevitably leads to corneal indentation from the ultrasound probe, and artificially shortened AL readings [27].

According to previous studies [29,30], AL measurements using US A-scan and optical biometers in eyes with cystoid macular edema differ significantly. Ueda et al. [29] evaluated the relationship between the difference in AL measurements with US A-scan and partial coherence laser interferometry and macular retinal thickness in patients with macular edema. They found positive correlation between AL intermethod difference and retinal thickness, if retinal thickness was more than 200 microns. This is likely a result of the fundamentally different methodology of the two device in measuring eyes with a pathologically thickened retina [29,30]. Conventional US A-scan measures the distance between the anterior corneal apex and the internal limiting membrane of the retina, whereas the optical AL is measured from the anterior corneal surface to the central peak of retinal pigment epithelium instead of the vitreoretinal interface, meaning that any change in retinal thickness (such macular edema) will not affect the overall AL measurement.

In previous studies [9-19,21], shorter ocular ALs in RVO eyes were measured by A scan US, and these results may due to macular edema complications of RVO [20]. Therefore in our study we choose the OLCR device in order to avoid measurement altering effects of macular edema.

Moghimi et al. [20] studied 29 patients with unilateral CRVO by partial coherence laser interferometry and did not find any significant difference in AL comparing affected eyes with unaffected fellow eyes. They described a statistically significant shorter posterior segment length (which was calculated as AL minus ACD) in affected eyes, although they did not have control eyes of unaffected patients. The role of shorter posterior part of the eye in CRVO was first emphasized by Moghimi et al., but they did not study the lens thickness and the vitreous chamber depth, therefore the exact structural correlate could not be determined.

In our study besides AL, other biometrical parameters (CCT, ACD, LT) of eyes in patients with RVO and age- and gender-matched control group were measured, allowing us to determine that CCT, ACD and LT of the affected eyes, unaffected fellow eyes, and control eyes did not differ statistically in patients with either CRVO or BRVO. We observed significantly shorter AL in the affected eyes of patients with CRVO and BRVO compared with control eyes. Mean CCT, ACD and LT of the affected eyes, unaffected fellow eyes and control eyes did not differ statistically either in patients with CRVO or BRVO. Therefore the finding that AL was significantly shortened in CRVO and BRVO affected eyes compared with controls can be attributed to a shorter VCD.

Using ultrasound, multiple authors have reported significantly shorter AL in the affected eyes of patients with CRVO [9,14,17,21] and BRVO [15-17] compared with unaffected fellow eyes. It was argued that significant differences between AL in RVO eyes and unaffected fellow eyes could be due to the effect of macular edema in AL measurements [9,20].

In our study, both eyes in patients BRVO were significantly shorter than control eyes, however we did not find any significant difference in AL between affected and unaffected fellow eyes in patients with BRVO. Evidence on symmetry between two eyes of the same person has been reported previously, with Rajan et al. [31] reporting that 76% of patients had less than a 0.3 mm interocular difference in AL, and Jabbour [32] reporting a mean difference of 0.0028 ± 0.24 mm in AL between eyes. In our study the lack of significant difference between the AL of the affected and unaffected fellow eyes in BRVO patients is consistent with reports of normal interocular symmetry. In the CRVO group however, the mean AL of affected eyes was significantly shorter than those of the unaffected fellow eyes. These results support evidence that CRVO develops less frequently in both eyes than BRVO, with estimates that 1.4% of CRVO cases developed CRVO in the fellow eye over a 3-year period [30] compared with BRVO which developed in the fellow eye in 4.5-9% of BRVO cases [33,34].

According to previous histopathological studies thrombus formation was observed at or near the lamina cribrosa in eyes with CRVO [35] and at the arteriovenous junction in BRVO [36]. The sieve-like structure of the lamina cribrosa which is formed from interweaving fascicles of collagen bundles with no elasticity restricts the expansion of the vessels passing through it [9]. The thickness of the lamina cribrosa and peripapillary sclera adjacent to the optic nerve scleral canal increase significantly with decreasing AL [37]. Furthermore, shorter AL is associated with decreased disc area in normal subjects [38].

Cekic et al. [14] suggested that eyes with shorter AL may be predisposed to greater crowding of the central retinal vein and artery at the lamina cribrosa, and are therefore more likely to develop CRVO. It is postulated that shorter eyes have a smaller disc and a narrower scleral canal through which the retinal vessels are more tightly confined. This may reduce the blood flow in the vein and may lead to increased blood flow turbulence that could cause endothelial damage and thrombus formation at the lamina cribrosa and at further arteriovenous crossings [13,21]. This theory of anatomical restriction in the posterior pole of shorter eyes is supported by our findings of shorted AL and VCD in eyes with BRVO and CRVO when compared with control eyes.

There are several limitations of our study. Firstly, we evaluated only the presence of hypertension and diabetes mellitus in our subjects. Other systemic risk factors such as hyperlipidemia, cardiovascular diseases, blood hyperviscosity and diseases with hypercoagulation were not studied. Secondly, the limitation of optical biometry is its inability to measure through dense cataracts and other media opacities that obscure the macula, therefore patients with significant ocular media opacities were not enrolled.

## Conclusion

To our knowledge this is the first study using optical low coherence reflectometry that found significant association between shorter axial length, vitreous chamber depth and retinal vein occlusion. Other anterior segment parameters (K_average_, CCT, ACD, LT) did not differ significantly in affected, unaffected fellow and control eyes. Difference in axial length between affected and control eyes is a result of shorter vitreous chamber depth along the visual axis in patients with RVO. Our results suggest that besides other risks, shorter axial and vitreous chamber depth might be an additional potential anatomical predisposing factor for the development of retinal vein occlusion.

In contrast to previous studies using US devices measurements performed with OLCR were not affected by the presence of macular edema.

## Abbreviations

CRVO: Central retinal vein occlusion
BRVO: Branch retinal vein occlusion
HRVO: Hemispheric retinal vein occlusion
OLCR: Optical low coherence reflectometry
AL: Axial length
CCT: Central corneal thickness
ACD: Anterior chamber depth
LT: Lens thickness
VCD: Vitreous chamber depth
K_average_: Average keratometric power
SER: Spherical equivalent refraction
n: Number
US: Ultrasonography
PCLI: Partial coherence laser interferometry
NS: Not studied
ANOVA: One-way analysis of variance test
